# M. Delpech on Impeforate Os Uteri

**Published:** 1830-10-01

**Authors:** 


### XLIX.

### HOPITAL DE MONTPELLIER.

### M. Delpech on Imperforate Os Uteri.

The following case and accompanying observations are contained in the Mémorial des Hôpitaux du Midi, for March of the present year.

*Case.* Margaret G. ætatis 22, was brought by her parents to M. Delpech. She was much emaciated, and extremely feeble; complained of thirst, and had some pyrexia. In the hypogastric region was a rounded, moveable tumour, painful to the touch, and rising to about the level of the umbilicus; it created most uneasiness at the monthly periods, when it seemed to increase in volume, and fits of hysteria came on; its pressure on the bladder frequently impeded the flow of urine, and occasioned a fallacious desire of micturition. On examination per vaginam a projection was felt, which was clearly a portion of the tumour, and it was also distinguished by the finger in the rectum; at the bottom of the vagina, as well as in the hypogastrium, fluctuation could be felt with some attention, but no trace of the neck of the uterus or os tincæ existed. The patient had never menstruated.

When young she had enjoyed good health, and even possessed considerable muscular strength. The preceding symptoms had been coming on for the last six years, and on two occasions she had been attacked with violent pain in the belly, attended with serious fever.

M. Delpech had no doubt that the tumour was produced by retention of the menses in the uterus, and increased growth of the latter. After trying various means without success, the surgeon determined to open into the uterus and evacuate its contents. He procured a trocar seven inches in length and five lines in diameter, slightly and uniformly curved, and its canula perforated with several lateral openings in the upper third.

The patient being placed as in the operation for lithotomy, pressure was made on the hypogastric tumour, in order to render the vaginal portion more prominent and fixed. The fore and middle fingers were then introduced into the vagina, and made to touch the tumour, whilst the canula carrying an œsophagus bougie was guided along the fingers, until the extremity also touched a favourable part of the tumour. The bougie was then withdrawn, and the trocar introduced through the canula into the tumour. On its withdrawal a pint and a half of brown, inodorous, and oily-looking matter was discharged, when the hypogastric tumour was quickly reduced to half its former size. The same matter continued to flow slowly for some time, but had ceased by the fourth day after the operation. A moderate mucous secretion followed, the volume of the tumour still farther decreased, and although much induration remained, there was no irregularity to be discovered. On the twentieth day after the performance of the operation the menses appeared, and lasted for six days. Pains in the hypogastrium and a paroxysm of hysteria succeeded, but these appeared to be rather the force of habit, than dependent on any existing disease. In another month the menses flowed again, and the uterus was now no larger than it commonly is three months after parturition.

M. Delpech remarks that much difference exists between the obstruction of the os uteri from inflammation, and the congenital imperforation. According to the Professor there is infinitely more difficulty in keeping the opening pervious in the former than the latter, and more danger also in performing the operation. M. Delpech mentions the best mode of ascertaining a collection of fluid in the enlarged uterus, which consists in introducing two fingers of the left hand into the vagina, and resting them on the portion of the tumour which projects there, whilst the right hand is laid on the hypogastrium. Between the two fluctuation may be discerned. The Professor might have added, that this mode of examination is well adapted to all uterine tumours of any degree of magnitude. Poising the os uteri on the fingers of one hand the hypogastric tumour is pressed down with the other, which furnishes a very useful method of examination.

M. Delpech observes that the best mode of operating is by using a long and large curved trocar, introduced in the line of the pelvic axis, and by not allowing all the fluid to escape by the canula at first. A certain quantity remaining behind, continues to flow gradually through the opening and obviates the necessity of a tent or other dilating medium. The point to be perforated should be the most dependent. In support of these statements, M. Delpech remarks that the operation of puncturing for imperforate anus almost always succeeds without any further measures to maintain the opening. Is this so universally the case? At all events, M. Delpech protests against the use of tents or bougies after this operation, condemning them as not only unnecessary but pernicious. In the obstruction of the os uteri from inflammation, it is best to use a speculum, and make a crucial opening with a bistoury. The edges of the opening should be removed with the same instrument, in order that there may be a loss of substance, instead of a simple puncture, which is all that is necessary in the congenital imperforation.

Such are the opinions of M. Delpech. Practitioners see so few of these cases that the experience of the Professor is more valuable on this, than on ordinary points. We think, and have long thought, that cases of obstructed catamenia are generally treated in too routine a fashion, and that mechanical obstruction in the vagina or os uteri would be oftener found, if more frequently looked for.

